# DAF‐16 stabilizes the aging transcriptome and is activated in mid‐aged *Caenorhabditis elegans* to cope with internal stress

**DOI:** 10.1111/acel.12896

**Published:** 2019-02-17

**Authors:** Shang‐Tong Li, Han‐Qing Zhao, Pan Zhang, Chung‐Yi Liang, Yan‐Ping Zhang, Ao‐Lin Hsu, Meng‐Qiu Dong

**Affiliations:** ^1^ School of Life Sciences Tsinghua University Beijing China; ^2^ Peking University‐Tsinghua University‐National Institute of Biological Sciences (PTN) Joint Graduate Program Beijing China; ^3^ National Institute of Biological Sciences Beijing China; ^4^ Research Center for Healthy Aging China Medical University Taichung Taiwan; ^5^ Institute of Biochemistry and Molecular Biology National Yang‐Ming University Taipei Taiwan; ^6^ Department of Internal Medicine, Division of Geriatric and Palliative Medicine University of Michigan Ann Arbor Michigan; ^7^ Tsinghua Institute of Multidisciplinary Biomedical Research, Tsinghua University Beijing China

**Keywords:** aging, *Caenorhabditis elegans*, DAF‐16, proteostasis, stress response, transcriptional regulation

## Abstract

The roles and regulatory mechanisms of transcriptome changes during aging are unclear. It has been proposed that the transcriptome suffers decay during aging owing to age‐associated down‐regulation of transcription factors. In this study, we characterized the role of a transcription factor DAF‐16, which is a highly conserved lifespan regulator, in the normal aging process of *Caenorhabditis elegans*. We found that DAF‐16 translocates into the nucleus in aged wild‐type worms and activates the expression of hundreds of genes in response to age‐associated cellular stress. Most of the age‐dependent DAF‐16 targets are different from the canonical DAF‐16 targets downstream of insulin signaling. This and other evidence suggest that activation of DAF‐16 during aging is distinct from activation of DAF‐16 due to reduced signaling from DAF‐2. Further analysis showed that it is due in part to a loss of proteostasis during aging. We also found that without *daf‐16*, dramatic gene expression changes occur as early as on adult day 2, indicating that DAF‐16 acts to stabilize the transcriptome during normal aging. Our results thus reveal that normal aging is not simply a process in which the gene expression program descends into chaos due to loss of regulatory activities; rather, there is active transcriptional regulation during aging.

## 1 INTRODUCTION

Aging is characterized by a gradual deterioration of physiological functions and an increase in the probability of death. Old age is the single most important risk factor for all gerontological diseases from arthritis to Alzheimer's. Undoubtedly, aging is a process accompanied by numerous biological changes at different levels, from the molecular to the systemic and organismal levels (López‐Otín, Blasco, Partridge, Serrano, & Kroemer, 2013). However, the aging process has not been characterized comprehensively and quantitatively. As such, for practical reasons, aging is measured not by a metric of the process but by its endpoint—death. Surely, lifespan assay is a convenient way to assess the average rate of aging, but it does not inform anything about the dynamics.

The nematode *Caenorhabditis elegans* has long established itself as an ideal model for aging research. Key genetic pathways that regulate aging have been discovered originally in *C. elegans* and later confirmed in other organisms including mammals, and one of which is the insulin/insulin‐like growth factor 1 (IGF‐1) signaling pathway, or IIS (Kenyon, Chang, Gensch, Rudner, & Tabtiang, 1993). In the *C. elegans* IIS pathway, the sole insulin/IGF‐1 receptor DAF‐2 negatively regulates the FOXO transcription factor DAF‐16 through a cascade of kinases composed of PI3K/AGE‐1, PDK/PDK‐1, and AKTs. Phosphorylation by AKTs prevents DAF‐16 from entering the nucleus to regulate downstream target genes. Loss‐of‐function mutations of *daf‐2* or the downstream kinases extend lifespan in a *daf‐16‐*dependent manner (Dorman, Albinder, Shroyer, & Kenyon, 1995; Kenyon et al., 1993; Paradis, Ailion, Toker, Thomas, & Ruvkun, 1999).

The *C. elegans* aging process has been characterized to some extent, including behavioral declines at the organismal level, gross morphological changes (e.g., length, texture; Pincus, Smith‐Vikos, & Slack, 2011), fine structural changes (Herndon et al., 2002; McGee et al., 2011), and molecular changes (Budovskaya et al., 2008; Golden & Melov, 2004; Lund et al., 2002). Among the molecular changes that are fundamental to other types of changes occurring in the aging process, gene expression changes are relatively easy to measure, thanks to the microarray technology in the past and deep sequencing at present. Previously, by analyzing the *C. elegans* transcriptome on adult days 1, 3, 5, and 10 at 20°C, Rangaraju *et al.* identified transcriptional drift, which refers to loss of stoichiometry between mRNAs of the same functional group and loss of youth‐associated co‐expression patterns in aging worms, as an age‐associated degenerative process. Slowing down aging by knocking down *daf‐2* restricted the progression of transcriptional drift, arguing that transcriptional drift is closely related to aging (Rangaraju et al., 2015). However, it is not known how IIS contributes to the normal aging process. A recent study has found that the amount DAF‐2 protein increases from adult day 1 through day 10 (Tawo et al., 2017), but it is still unclear whether there is a decrease in transcriptional activity of *daf‐16* in the normal aging process, and whether there is, how much it contributes to transcriptional drift in aging *C. elegans*.

A few studies have suggested a possible link between transcriptome deterioration and age‐associated inactivation of transcription factors. One study showed that deactivation of a GATA factor *elt‐2* is responsible for age‐associated down‐regulation of more than 200 genes in wild‐type (WT) worms assayed on adult days 2, 5, 8, and 11 at 25°C (Mann, Van Nostrand, Friedland, Liu, & Kim, 2016). *elt‐2* plays an essential role in the development of the intestine, indicating that it is selected under strong selective pressure during evolution. Loss of the *elt‐2* transcriptional network after *C. elegans* has reached adulthood is consistent with the idea that transcriptional drift plays a role in age‐associated physiological deterioration. In comparison with that of *elt‐2*, age‐dependent repression of *hsf‐1* is strikingly early—within the first 12 hr of adulthood at 20°C, which is closely connected to the collapse of proteostasis during aging (Labbadia & Morimoto, 2015).

In this study, prompted by the observation of nuclear accumulation of DAF‐16 in mid‐aged *C. elegans *adults, we carried out mRNA‐Seq analysis of WT and *daf‐16 (null)* mutant worms every 24 hr from adult day 1 through day 7. Specifically, we set out to address three questions. First, whether DAF‐16 is activated during aging, and when? Second, what activates DAF‐16 during aging? Third, what is the role of DAF‐16 during normal aging? Through the mRNA‐Seq and follow‐up experiments, we verified that DAF‐16 is indeed activated during the aging process of WT *C. elegans*, most prominently on adult day 6 and day 7 at 25°C. Remarkably, the transcriptional output of DAF‐16 activated by age is much different from that by reduced signaling from DAF‐2. Improving proteostasis during aging delays DAF‐16 nuclear accumulation, suggesting that DAF‐16 is activated in response to a loss of proteostasis and possibly other types of age‐associated cellular stress. Lastly, we find that DAF‐16 acts as a “capacitor” to resist age‐induced perturbation of the gene expression program in the normal aging process.

## 2 RESULTS

### 2.1 DAF‐16 is activated in the normal aging process

**Figure 1:**
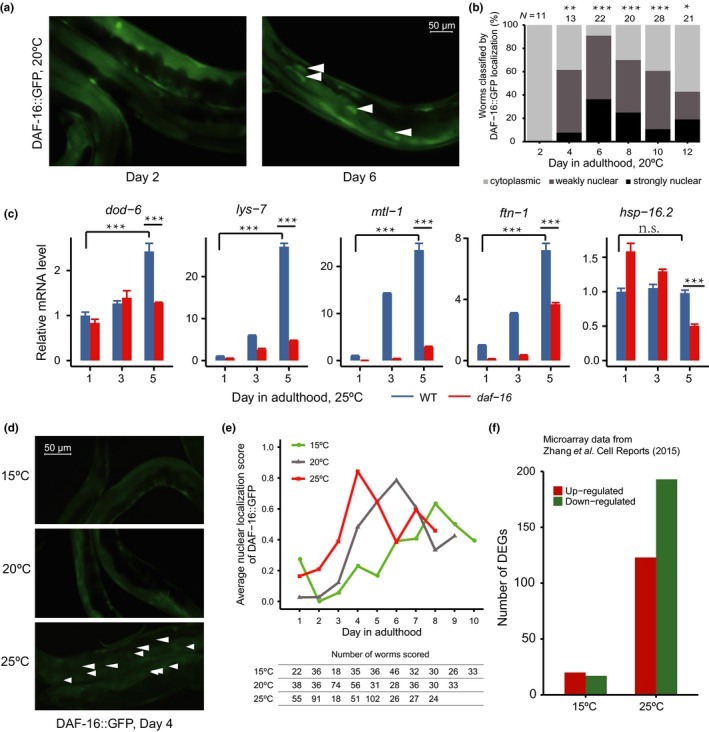
Activation of DAF‐16 during the normal aging process. (a and b) Age‐dependent nuclear accumulation of DAF‐16::GFP in wild‐type *Caenorhabditis elegans*. Representative images (a) and the quantitation result (b) are shown; the Fisher exact test was performed for DAF‐16::GFP localization comparing day 2 with other time points. More images are shown in Supporting Information Figure S1. *p < 0.05; **p < 0.01; ***p < 0.001; Fisher exact test, day 2 v.s. others. (c) Quantitative RT‐PCR analysis of known DAF‐16 target genes, expressed as mean ± standard error (*n* = 3). *p < 0.05; **p < 0.01; ***p < 0.001; Student's *t* test. (d and e) An increase in culture temperature accelerates DAF‐16 nuclear accumulation. Representative images and the quantitation result are shown in (d) and (e), respectively. The nuclear localization score is described in Experimental Procedures. On day 4, *p* < 0.05, 25°C versus 20°C; *p* < 0.01, 25°C versus 15°C; Fisher exact test. (f) Re‐analysis of published microarray data (Zhang et al., 2015). Plotted are the numbers of DEGs between wild‐type and *daf‐16(mgDf47) *worms on adult day 3 at either 15°C or 25°C

While observing WT worms expressing a DAF‐16::GFP fusion protein under standard, nonstressful conditions (20°C, well‐fed), we noticed nuclear accumulation of DAF‐16::GFP in older animals but not in the young ones (Figure 1a and Supporting Information Figure S1). Quantification of the localization patterns of DAF‐16::GFP verified this observation (Figure 1b). At 20°C, DAF‐16::GFP translocated into the nucleus starting from adult day 4. As nuclear localization typically represents DAF‐16 activation (Henderson & Johnson, 2001; Lee, Hench, & Ruvkun, 2001; Lin, Hsin, Libina, & Kenyon, 2001), this result suggests that DAF‐16 is activated during the normal aging process. Supporting this idea, quantitative real‐time PCR (qRT‐PCR) analysis showed that four out of five known DAF‐16 target genes showed higher mRNA levels in day‐3 and day‐5 WT adults than in day‐1 adults, and this was dependent on *daf‐16* (Figure 1c). As a poikilothermic animal, *C. elegans* ages faster at higher temperatures. If DAF‐16 activation is driven by aging, one would predict accelerated (or decelerated) DAF‐16::GFP nuclear accumulation in worms grown at 25°C (or 15°C) compared to those at 20°C. This is indeed the case (Figure 1d and e). We also noticed that near the end of the observation period, which lasted for 8–10 days, the extent of DAF‐16::GFP nuclear accumulation lessened somewhat. The microscopy result (Figure 1e) suggests that on adult day 3, there is substantial nuclear accumulation, and presumably activation, of DAF‐16 in WT animals grown at 25°C, but not at 15°C. This is validated by re‐analysis of the microarray data of day‐3 WT and *daf‐16(null)* adults cultured at 15°C or 25°C (Zhang et al., 2015), which identified 316 differentially expressed genes (DEGs) between WT and *daf‐16(null)* at 25°C, but only 37 DEGs at 15°C (Figure 1f).

### 2.2 Effect of DAF‐16 activation on age‐associated gene expression changes

**Figure 2:**
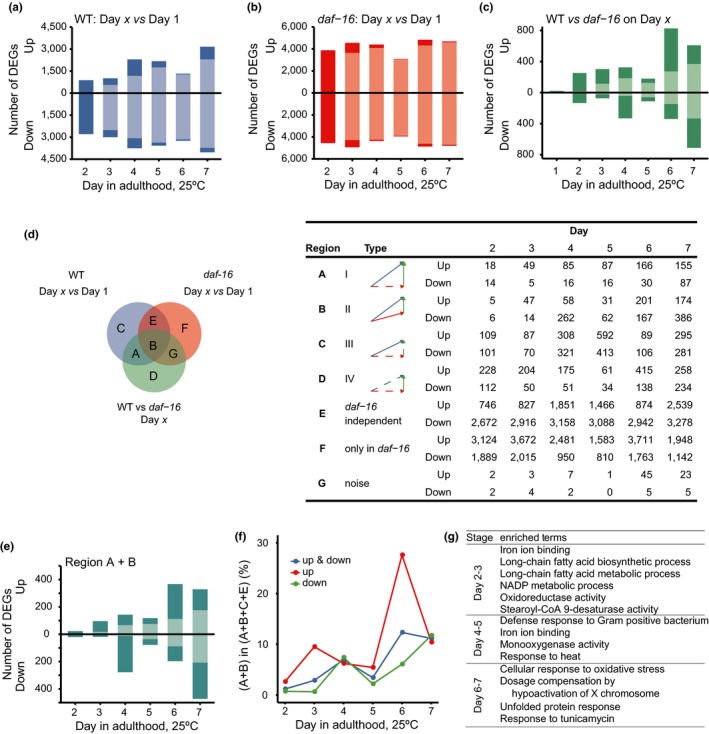
Transcriptome analysis of WT and *daf‐16(null) *adults from day 1 through day 7. (a) Number of genes that are up‐ or down‐regulated in WT on day *x* relative to day 1. Darker shades, DEGs detected for the first time; lighter shades, DEGs detected at a previous time point. (b) Number of genes that are up‐ or down‐regulated in *daf‐16(mu86) *worms on day *x* relative to day 1. (c) Number of genes that are up‐ or down‐regulated in WT relative to the *daf‐16(mu86) *mutant on each day from day 1 through day 7. (d) A mock Venn diagram to illustrate the subsets of DEGs according to how they are affected by age and by *daf‐16(mu86)* (left) and the number of DEGs of each subset on each day (right). A blue arrow denotes a change between Day 1 and Day *x* for WT, and a red one for the *daf‐16* mutant. A green arrow denotes the difference between WT and the *daf‐16 *mutant on Day *x*. Statistically significant differences are indicated by solid arrows, otherwise by dashed arrows. (e) Number of age‐ and DAF‐16‐dependent DEGs (Region A + B) on Day *x* relative to Day 1. (f) Percentage of age‐ and DAF‐16‐dependent DEGs at the indicated day of age for WT* Caenorhabditis elegans*. This is calculated as (Region A + B)/(Region A + B + C + E) × 100%. (g) Enriched GO terms among the up‐regulated genes in Region A + B. Corrected *p*‐value <0.05, fold enrichment >2

To fully characterize the phenomenon of DAF‐16 activation during aging, we performed mRNA‐Seq analysis of WT and *daf‐16(mu86) *null mutant worms at high temporal resolution. Samples were collected every 24 hr from adult 1 through day 7 at 25°C. The *fer‐15(b26ts)* allele, which causes temperature‐sensitive sterility but does not affect lifespan, was used in the background to avoid sample contamination by progeny. For WT worms, using adult day 1 as reference, we found that the number of DEGs increased over the next 6 days (Figure 2a). For *daf‐16(mu86)* null animals, the increase in DEGs was much more abrupt (Figure 2b). To find out which of the genes showing age‐associated expression changes were dependent on *daf‐16*, we compared the transcriptomes of WT and *daf‐16(null)* worms from day 1 through day 7 (Figure 2c). As shown, there are few DEGs between WT and *daf‐16(null)* on day 1, confirming that DAF‐16 activity in WT worms is inhibited, which is consistent with the cytoplasmic distribution pattern of DAF‐16::GFP on day 1. We defined age‐ and *daf‐16‐*dependent DEGs for each day from day 2 to day 7 as those in Regions A and B in the Venn diagram illustration in Figure 2d, which increased from 43 on day 2 to 802 on day 7 (Figure 2d,e). The daily percentage of them among age‐dependent DEGs followed a similar trend rising from 1.2% on day 2 and peaking at 12.3% on day 6 (Figure 2f). Besides the DEGs in Regions A and B, those in C and D are also *daf‐16* dependent in some aspects. Comparing the transcriptome of WT on day *x* relative to that of either WT on day 1 or the *daf‐16* mutant on day *x*, we found that the Spearman's correlation increased steadily from 0.03 on day 2 to 0.46 on day 7, suggesting that the *daf‐16* gene activity contributes more and more to age‐associated transcriptome remodeling (Supporting Information Figure S2). These mRNA‐Seq results are consistent with DAF‐16 activation during aging as indicated by DAF‐16::GFP nuclear accumulation (Figure 1).

The mRNA‐Seq analysis also revealed that DAF‐16 plays a role in stabilizing the transcriptome against perturbations caused by aging. Without *daf‐16*, adding only one day of age on top of day 1 was enough to change the expression of 8,446 genes, whereas in the presence of *daf‐16*, it took five more days to reach 7,195 DEGs (Figure 2a,b). On each day after day 1, we found thousands of DEGs unique to the *daf‐16* mutant (Region F), greatly outnumbered the DEGs unique to the wild type (Regions A + C; Figure 2d).

For nearly all the DEGs uniquely found in the *daf‐16* mutant (Region F), their expression levels also increased or decreased in the WT after day 1, but not statistically significant enough to be classified as DEGs in the WT. For all genes, the fold change of mRNA reads on day *x* relative to day 1 varied to a greater extent in the *daf‐16* mutant than in the WT (Supporting Information Figure S3).

In conclusion, the *daf‐16 *gene activity seems to fulfill the function of a “capacitor” for thousands of genes to resist age‐associated transcriptional alterations. This function was not known before, and it contrasts intriguingly with the regulator function of *daf‐16*, which activates or represses the expression of a few hundred genes after day 1 (Regions A + B). The regulator function of *daf‐16* directly shapes the age‐dependent expression profile of WT, but the capacitor function is hidden from view until *daf‐16* is deleted.

### 2.3 The age‐dependent DAF‐16 targets are not the same as the classic IIS‐dependent DAF‐16 targets

**Figure 3:**
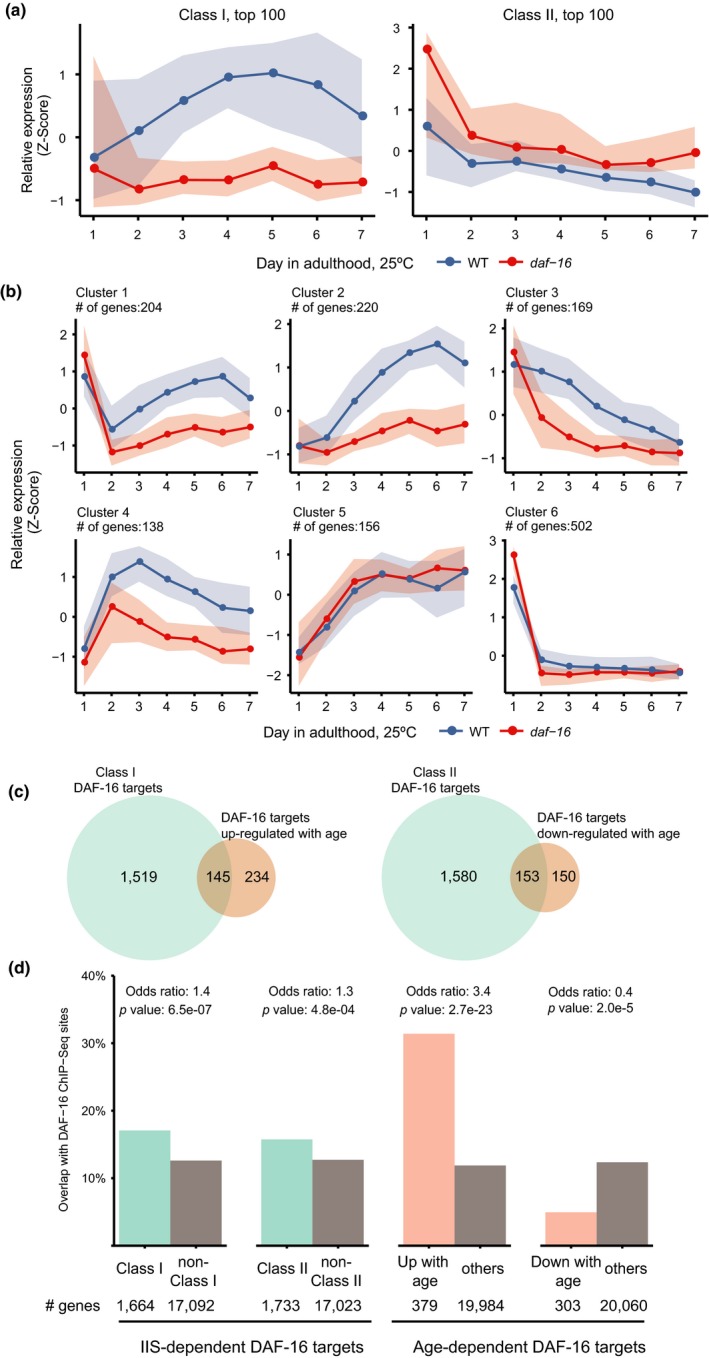
Genes regulated by DAF‐16 during aging are different from the classic insulin signaling targets. (a) Relative expression (*Z*‐Score) of the most prominent IIS targets in the first week of adulthood. Left panel: top 100 Class I genes; right panel: top 100 Class II genes. (b) *k*‐means clustering of the temporal expression profiles of Class I genes. For both (a) and (b), the lines and the shade boundaries indicate the 50th, 20th, and 80th percentiles. The expression levels of each gene across all samples are centered to the mean and scaled to the variance. (c) Overlap of the classic IIS targets and the genes regulated by DAF‐16 during aging. (d) Overlap of the classic IIS targets and the genes regulated by DAF‐16 during aging with DAF‐16 ChIP‐Seq binding sites

One immediate question about age‐associated activation of DAF‐16 is whether this is caused by a gradual reduction of IIS. To find out the answer, we looked into the expression profiles of the IIS targets during aging. The IIS targets, consisting of 1,663 genes that are up‐regulated (Class I) and 1,733 genes that are down‐regulated (Class II) upon DAF‐16 activation, have been defined by comparing the *daf‐2* mutant and the *daf‐2; daf‐16* double mutant at the beginning of adulthood (Tepper et al., 2013). If IIS decreases with age, the expression of Class I genes should increase and that of Class II genes should decrease with age in a *daf‐16*‐dependent manner. By examining the collective temporal expression profiles of the most highly ranked class I and class II genes, we found that as WT worms grow old, the top 100 class I genes tend to be up‐regulated in a *daf‐16*‐dependent manner, but their class II counterparts are down‐regulated independently of *daf‐16* (Figure 3a). Further analysis of all Class I genes by *k*‐means clustering showed that although 424 genes in clusters 1 and 2 support the idea of reduced IIS during aging, the other four clusters, containing nearly 1,000 genes, do not (Figure 3b). Collectively, these results show that DAF‐16 activation during aging cannot be simply explained by either activation or inactivation of IIS.

A close examination of the mRNA reads of insulin‐like (INS) peptides supported the above conclusion. Of the 22 *ins* genes quantified, *ins‐4*, *ins‐6*, *ins‐7*, *ins‐17*, *ins‐22*, *ins‐23*, *ins‐26*, *ins‐27*, *daf‐28*, and *daf‐31 *are relatively high‐confidence DAF‐2 agonists (Fernandes de Abreu et al., 2014), but their mRNA levels did not decrease with age in the WT; *ins‐1* and *ins‐3* are two relatively high‐confidence DAF‐2 antagonists (Fernandes de Abreu et al., 2014), but their mRNA levels did not increase with age (Supporting Information Figure S4). For the other *ins* mRNA quantified, it remains unclear whether they encode agonists or antagonists, but none except *ins‐18* and *ins‐19* showed obvious age‐associated expression changes in the WT (Supporting Information Figure S4).

To better characterize the age‐dependent DAF‐16 target genes, we defined them as those whose expression levels were significantly higher or lower in the WT than in the *daf‐16(mu86)* mutant on both day 6 and day 7 when the number of DEGs rose to a peak (Figure 2c), and on both days, the fold change of WT versus *daf‐16(mu86)* must be greater than that on day 1. This led to a total of 379 target genes that are up‐regulated by DAF‐16 with age and 303 that are down‐regulated by DAF‐16 with age. Not surprisingly, less than 10% of the classic IIS‐dependent DAF‐16 targets, either Class I or Class II, are age‐dependent DAF‐16 targets (Figure 3c, Supporting Information Table S1). Conversely, only 50% or less of the age‐dependent DAF‐16 targets were previously identified as IIS‐dependent DAF‐16 targets (Figure 3c).

Using the DAF‐16 ChIP‐Seq data produced by the ModEncode project (Gerstein et al., 2010), we found a 3.4‐fold enrichment of the in vivo DAF‐16 binding sites among the target genes activated by DAF‐16 on adult days 6–7, and a remarkable 2.5‐fold depletion of these sites among the targets repressed by DAF‐16 (Figure 3d). In contrast, the same in vivo DAF‐16 binding sites were enriched moderately (1.3‐ or 1.4‐fold) among the IIS‐dependent DAF‐16 targets of either Class I (activated by DAF‐16) or Class II (repressed by DAF‐16; Figure 3d). This again suggests that IIS is unlikely the only mechanism that governs DAF‐16 activation during aging.

Echoing the same conclusion, KEGG analysis of the IIS‐dependent DAF‐16 targets and the age‐dependent DAF‐16 targets uncovered a unique set of KEGG terms that are enriched in the latter. Most intriguingly, genes involved in mTOR signaling or ErbB signaling are specifically enriched among the targets up‐regulated by DAF‐16 on days 6–7 (Figure S5b). KEGG term enrichment analysis results of other categories of genes with age‐dependent expression changes are shown in Supporting Information Figure S6.

### 2.4 Activation of DAF‐16 in response to age‐associated proteostasis collapse

**Figure 4:**
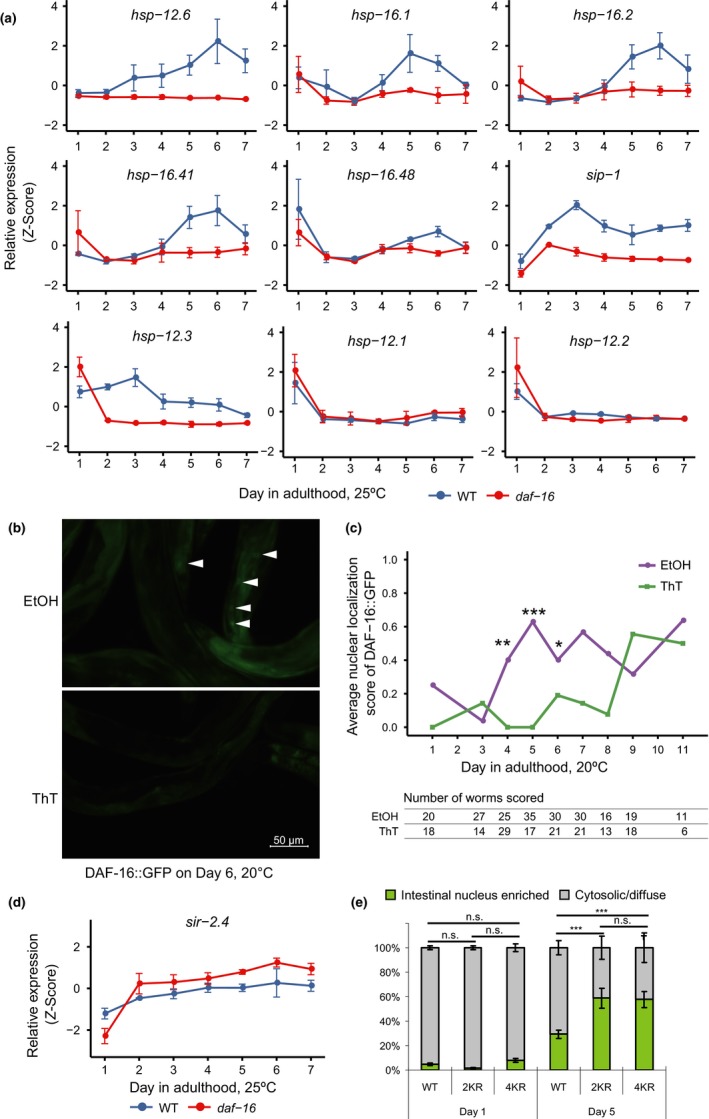
Activation of DAF‐16 during aging is connected to the loss of proteostasis. (a) Temporal expression profiles of the genes encoding small heat‐shock proteins. (b and c) Thioflavin T (ThT) attenuated age‐dependent DAF‐16 nuclear localization. Representative images (b) and the quantitation result (c) are shown. *p < 0.05; **p < 0.01; ***p < 0.001; ThT v.s. EtOH on the same day, Fisher exact test. (d) The temporal expression profile of *sir‐2.4.* (e) DAF‐16 K to R mutations, which abolish acetylation and sensitize DAF‐16 for nuclear translocation, enhanced age‐dependent nuclear accumulation of DAF‐16::GFP. Data were analyzed from three independent biological repeats, and the *p*‐values were calculated using chi‐square test. *N* > 60

Next, we tried to find out what may be the cause of DAF‐16 activation in the normal aging process. Besides IIS, DAF‐16 can be regulated by other signals in the cellular environment, including various stress signals such as heat, oxidative stress, and starvation (Henderson & Johnson, 2001). Since different types of stress are typically coupled together during the aging process, discerning cause and consequence remains the most challenging in aging research. In this study, we focused on the collapse of proteostasis as it is shown to be an early and profound aging phenotype in *C. elegans* and is proposed to be a driving force of aging (Ben‐Zvi, Miller, & Morimoto, 2009). The collapse of proteostasis induces the expression of various heat‐shock proteins (HSPs), which help refold damaged proteins. From our mRNA‐Seq data, we found evidence of *daf‐16*‐dependent induction of multiple small HSPs a few days into the adulthood (Figure 4a).

Additionally, gene ontology (GO) analysis of age‐ and DAF‐16‐dependent DEGs have suggested that among the up‐regulated DEGs identified from day 4 to day 7, a number of stress response terms are significantly enriched (Figure 2g). For example, response to heat and defense response to Gram‐positive bacterium is enriched most prominently on day 4 and day 5; unfolded protein response, cellular response to oxidative stress, and response to tunicamycin (ER stress) are enriched on day 6 and day 7.

This suggests that the collapse of proteostasis is a plausible cause of DAF‐16 activation in older worms particularly on day 6 and day 7. To test this hypothesis, we treated *C. elegans* with thioflavin T (ThT), a compound that improves proteostasis and extends lifespan by binding to and stabilizing amyloids (Alavez, Vantipalli, Zucker, Klang, & Lithgow, 2011). Indeed, ThT treatment delayed nuclear accumulation of DAF‐16::GFP by 5 days at 20°C (Figure 4b,c), suggesting that the collapse of proteostasis is a cause for DAF‐16 activation in older worms, although it may not be the only one.

SIR‐2.4, the *C. elegans* homolog of mammalian SIRT6 and SIRT7, promotes stress‐induced DAF‐16 nuclear localization by removing the acetyl modification groups from several lysine residues of DAF‐16 (Chiang, Tishkoff, et al., 2012). Our mRNA‐Seq experiments showed that the expression of *sir‐2.4* was elevated in older worms (Figure 4d). Induction of *sir‐2.4* is in favor of promoting DAF‐16 nuclear localization. Consistently, 2KR and 4KR mutations, both of which preclude CBP‐1‐dependent acetylation and thus sensitize DAF‐16, enhanced age‐dependent nuclear localization of DAF‐16::GFP (Figure 4e). To sum up, the evidence above strongly suggests a mechanism of DAF‐16 activation in response to age‐associated cellular stress.

### 2.5 Additional transcription factors cooperate with DAF‐16 to shape the aging transcriptome

**Figure 5:**
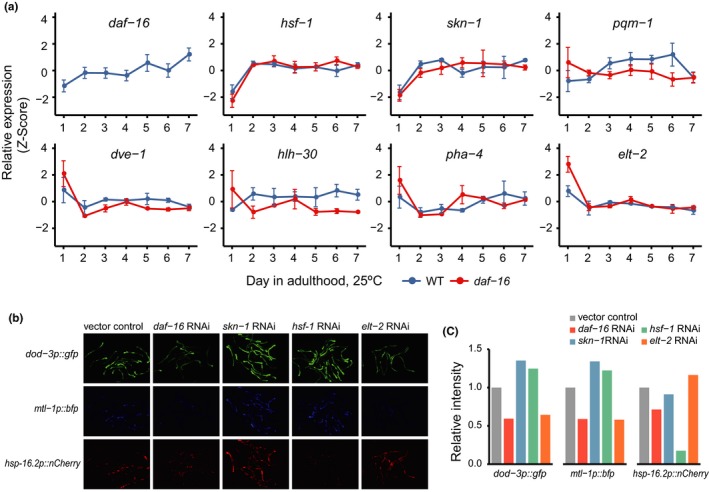
Additional Transcription factors cooperate with DAF‐16 to shape age‐dependent gene expression changes. (a) Expression profiles of *daf‐16* and seven additional transcription factors as seen in our mRNA‐Seq analysis. (b and c) Knocking down some of the transcription factors by RNAi affected the expression of *dod‐3*, *mtl‐1*, and *hsp‐16.2* on adult day 5 as indicated by fluorescent reporters. Representative images (b) and the quantitation result (c) are shown

Lastly, we asked what additional transcription factors besides DAF‐16 may take part in age‐associated transcriptome remodeling. The mRNA‐Seq analysis uncovered thousands of DAF‐16‐independent DEGs from day 2 to day 7, compared with day 1 (Region E, Figure 2d). Also, there are DEGs that are only partially dependent on DAF‐16 (Region B, Figure 2d). Expression of *daf‐16* itself increases with age (Figure 5a), which is likely a result of transcriptional activation by other transcription factors, because *daf‐16* is not a class I gene, that is, not a target of positive regulation by its own gene product (Tepper et al., 2013). Thus, there must be additional TFs that govern age‐dependent transcription. We examined the temporal expression profiles of seven additional TFs that have been shown to play a role in lifespan regulation (Figure 5a). In both WT and *daf‐16* mutant worms, expression of *hsf‐1* and *skn‐1* increased with age, while expression of *elt‐2* decreased with age. In contrast, the age‐dependent increase in *pqm‐1* expression was dependent on *daf‐16*, and so was that of *hlh‐30*. The temporal expression profiles of *pha‐4* and *dve‐1* were more complex. The expression profiles of some of these transcription factors’ target genes are shown in Supporting Information Figure S7. We wondered whether *hsf‐1* and *skn‐1* may cooperate with *daf‐16* during aging, because they are critical for stress response (An & Blackwell, 2003; Hsu, Murphy, & Kenyon, 2003) and their expression levels increase with age. Using the transcriptional reporter strains of *dod‐3*, *mtl‐1,* and *hsp‐16.2*, we found that *hsf‐1* is required for the enhanced expression of *hsp‐16.2p::nCherry* in mid‐aged worms, but not for that of *dod‐3p::gfp* and *mtl‐1p::bfp* (Figure 5b,c). *skn‐1* RNAi had no effect on all three. Six well‐characterized *skn‐1* target genes (*gst‐4/5/7/10/13/38*) (Oliveira et al., 2009; Tullet et al., 2008) are generally down‐regulated during aging (Supporting Information Figure S7), which is consistent with a previous finding (Ewald, Landis, Porter Abate, Murphy, & Blackwell, 2015) and thus indicates a loss of SKN‐1 transcriptional activity despite the increase in *skn‐1* mRNA. We also examined the effect of *elt‐2* RNAi, as *elt‐2 *has been reported to regulate insulin signaling targets and is responsible for age‐dependent down‐regulation of several hundred genes (Mann et al., 2016; Zhang, Judy, Lee, & Kenyon, 2013). Indeed, *elt‐2* was required for the induction of *dod‐3p::gfp* and *mtl‐1p::bfp* expression in mid‐aged worms (Figure 5b,c). Therefore, at least two additional TFs, ELT‐2 and HSF‐1, modulate gene expression changes with age. It remains to be known whether or not they interact with DAF‐16, and how.

### 2.6 Importance of DAF‐16 for wild‐type lifespan

**Figure 6:**
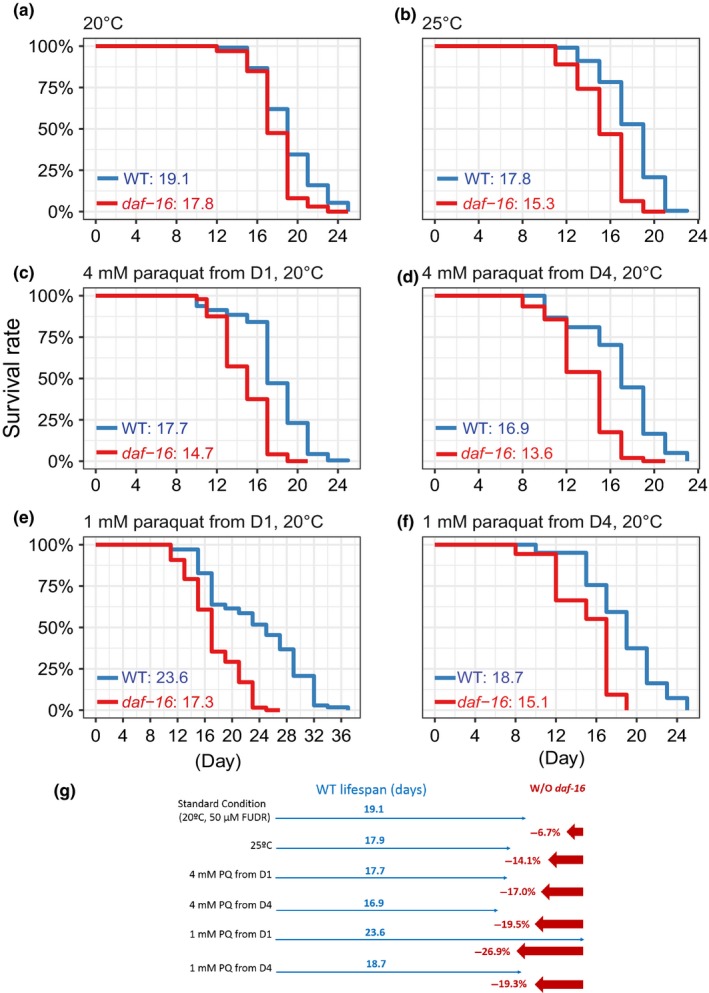
Lifespan analysis of WT and *daf‐16(null) *worms under the standard or stress conditions. (a to f) Lifespan curves of WT and *daf‐16(mu86)* under either the standard assay condition (a) or various conditions of mild stress (b–f). The mean lifespan values are indicated. The difference between the WT lifespan and daf‐16 lifespan is significant in each of the conditions assayed. *p* < 0.01 according to log‐rank test, *n* ≥ 59. (g) A summary of the assay results

The above results strongly suggest that DAF‐16 plays an important role during the normal aging process of *C. elegans*. However, the *daf‐16(null)* mutants live only slightly shorter than WT under the standard lifespan assay condition of 20°C, with plenty of OP50 food on NGM plates (Lin et al., 2001). As DAF‐16 is activated during aging in response to internal stress, we suspect that under the standard assay condition of minimal stress, the importance of DAF‐16 may be masked. We therefore carried out a set of lifespan assays under less‐favorable conditions by raising the temperature from 20°C to 25°C or by applying paraquat (PQ) at low concentrations starting from adult day 1 or day 4 (Figure 6, Supporting Information Table S2). None of these treatments shortened the WT lifespan by more than 2 days, and some even extended the WT lifespan due to a hormesis effect (Figure 6e, Supporting Information Table S2). As shown, *daf‐16(null)* reduced the WT lifespan by only 1.3 days or 6.8% under the standard assay condition. Under the less‐favorable conditions, *daf‐16(null)* reduced substantially the WT lifespan by 14.1% to 26.9% (Figure 6g). To be noted, the treatment of 1.0 mM paraquat from adult day 4 at 20°C did not affect the WT lifespan (*p* = 0.94), and under this condition, *daf‐16(null)* shortened WT lifespan by 19.3% (*p* < 0.001) (Figure 6a,f and g, Supporting Information Table S2). These results demonstrate that it takes only a tiny amount of stress to manifest the fact that DAF‐16 is indispensable for WT lifespan. Hence, DAF‐16 should be important for WT *C. elegans* living in the wild where stress is all too common.

## 3 DISCUSSION

### 3.1 DAF‐16 activation in mid‐aged worms indicates that aging is not entirely haphazard

Aging is intriguing. It seems inevitable for animals that age, even if they are given the best living conditions possible. One school of evolutionary theories including the widely known antagonistic pleiotropy theory attribute the origin of aging to a declining force of selection on individuals after reproduction begins (Trindade et al., 2013; Williams, 1957). The weakening or lacking of purifying selection eventually takes out proper regulation of a living system and lets mutations that have detrimental effects late in life accumulate in the genome. Based on these theories, one might expect the aging process to unfold in a passive and haphazard way. However, our high‐resolution transcriptome analysis of *C. elegans* adults at 25°C from day 1 through day 7 has depicted a different view. Activation of DAF‐16 is detectable by mRNA‐Seq starting from day 2 and becomes well pronounced after day 5 (Figures 1e and 2c, and Supporting Information Figure S2) when reproduction has ended and aging undoubtedly kicks in. Activation of DAF‐16 turns on the expression of many stress response genes as is evident from the significantly enriched GO terms (Figure 2g) such as defense response to Gram‐positive bacterium, response to heat, response to oxidative stress, unfolded protein response, and response to tunicamycin (ER stress). Thus, DAF‐16 is activated in the normal aging process for protection and repair, that is, anti‐aging. Previous studies have identified transcription factors such as ELT‐2 and ELT‐3 whose deactivation during aging drives the transcriptional network into disorder (Budovskaya et al., 2008; Mann et al., 2016). Here, by showing DAF‐16 activation after adult day 1, we provide evidence for active gene expression remodeling during normal aging. This shows that the aging process is not entirely passive. Also, based on the GO terms of the DAF‐16 targets that are turned on from day 2 to day 7, this period may be divided into three phases. The first one, of days 2–3, is significantly enriched for GO terms related to metabolism, especially fatty acid metabolism. This coincides with a remarkable increase in fatty acid species previously detected around the same period (Gao et al., 2017). The third one, of days 6–7, is significantly enriched for GO terms of stress response. In between is the transition phase (Figure 2g). Related to this, the genes regulated by DAF‐16 in each of three phases (Supporting Information Table S1) differ in the number (Supporting Information Figure S5a), the degree of overlap with the classic IIS targets (Supporting Information Figure S5a and Figure 3c), and in the enriched KEGG terms (Supporting Information Figure S5b).

Although it is difficult to know precisely when aging starts, because there is not a unanimous criterion, most if not all gerontologists would agree that aging starts no later than the end of reproduction. For *C. elegans* grown at 25°C, reproduction ends at the end of day 3. If day 4 is the start line, then aging starts with the transition phase followed by a stress response phase, and possibly others that have not been characterized. This shows that the aging process is not entirely haphazard; it proceeds in an orderly fashion at least in the beginning.

Taken together, our surprising finding of DAF‐16 activation during normal aging indicates that aging is not entirely a passive and haphazard process as often perceived before. There is active response to defend against internal stress brought about by old age, and there is order among disorder, at least in the early phase of aging.

### 3.2 Regulation of mTOR signaling by DAF‐16 during aging

By defining nearly 700 age‐dependent DAF‐16 targets (Figure 3c), we were surprised to find that more than half of them were not identified before as DAF‐16 targets downstream of IIS. KEGG analysis of the IIS‐dependent and age‐dependent DAF‐16 target genes revealed that genes involved in mTOR signaling are enriched among the targets up‐regulated by DAF‐16 during aging, but not among the IIS‐dependent DAF‐16 targets (Supporting Information Figure S5b). In mammalian cells, IIS regulates, and is regulated by, the mTOR pathway (Laplante & Sabatini, 2012). In *C. elegans,* both pathways critically regulate metabolism and aging, and there is cross‐talk between them (Chen et al., 2013; Seo et al., 2013), but no clear evidence has been found to place the mTOR pathway downstream of IIS. For example, the mTOR pathway genes are not known to be transcriptional targets of DAF‐16 in response to reduced DAF‐2 signaling. DAF‐16 binding sites have been identified in the promoter region of *rheb‐1*, but only in the *shc‐1* mutant background was DAF‐16 overexpression found to promote the expression of *rheb‐1*. Moreover, *daf‐16* was dispensable for the induction of *rheb‐1* in the *daf‐2* mutant (Qi et al., 2017 *)*. Our mRNA‐Seq analysis uncovered that components of mTOR signaling such as *unc‐51, dsh‐1, and mop‐25.1* are up‐regulated by DAF‐16 in older worms. Why these mTOR signaling genes become downstream targets of DAF‐16 only in aged worms awaits further investigation.

### 3.3 DAF‐16 activation in response to the loss of proteostasis

DAF‐16 is known to integrate multiple genetic and environmental stimuli (Kenyon, 2010), and this study uncovers aging as another one. DAF‐16 activation during aging produces a set of transcriptional outputs different from that by reduction of IIS. *C. elegans* and humans have a long postreproductive lifespan. In both systems, postmitotic cells live for a long time, either in absolute timing units (e.g., neurons and cardiomyocytes in humans) or relative to the length of the reproductive period (all somatic cells in *C. elegans*). Without the ability to dilute damaged proteins by cell division, little is known about how these cells handle the loss of proteostasis, a hallmark of aging (López‐Otín et al., 2013). In human, repressor element 1‐silencing transcription factor (REST), which is often lost in Alzheimer's patients, may be activated in healthy seniors to induce the expression of stress response genes and to repress the genes that promote cell death and Alzheimer's (Lu et al., 2014). This suggests that cellular stress response plays an active role in healthy aging. Consistently, this study finds that the loss of proteostasis is a cause of DAF‐16 activation during normal aging. Modulating proteostasis by temperature or amyloid‐binding compounds changes the timing of DAF‐16 nuclear accumulation. It has been shown that *hsf‐1*‐mediated heat‐shock response is repressed from early adulthood (Labbadia & Morimoto, 2015), so the stress response mediated by DAF‐16 in mid‐aged worms, which includes the induction of small HSPs, implicates an arms race between cellular protection mechanisms and aging.

### 3.4 Role of DAF‐16 in wild‐type *C. elegans*

The *daf‐16(null)* mutants live slightly shorter than WT on standard culture plates at 20°C (Lin et al., 2001). In comparison, loss of *daf‐16* produces a dramatic effect in the *daf‐2 *mutant background as *daf‐16(null)* completely abolishes the exceptional longevity of *daf‐2* mutant worms (Kenyon et al., 1993). Besides, unlike *daf‐2(null)* mutants, which are lethal, *daf‐16(null)* mutants are perfectly viable and fertile. This makes one wonder what purpose *daf‐16* serves in WT *C. elegans*. Here, we show that although DAF‐16 is largely inactive on adult day 1, judging by the fact that there is little difference between WT and *daf‐16(null)* at this point (Figure 2c), DAF‐16 acts as a “capacitor” to stabilize the transcriptome during aging (Compare Figure 2a and b). Probably, equally important is activation of DAF‐16 in response to age‐associated internal stress in older worms. Although *daf‐16* mutants appear to be WT‐like when there is little stress, such as under the standard lifespan assay condition, they live much shorter than WT when the stress level is elevated even by so slight an amount as not to affect the WT (Figure 6). For *C. elegans* living in the wild where encountering stress is the norm, DAF‐16 may be important for a normal lifespan.

### 3.5 Timing of DAF‐16 activation for longevity

Previously, an elegant genetic study has demonstrated that for the longevity phenotype induced by reduction of IIS, activation of *daf‐16* in early adulthood is the most critical (Dillin, Crawford, & Kenyon, 2002). In this study, we find that *daf‐16* is activated in mid‐aged WT animals. This is consistent with the timing requirement of lifespan regulation by IIS. It explains why late activation of DAF‐16 by reducing IIS is less effective—because DAF‐16 is already activated.

## 4 EXPERIMENTAL PROCEDURES

### 4.1 *C. elegans* strains

Strains used in this work include N2, CF1038 *daf‐16(mu86) I*, MQD54 *hqIs9[daf‐16p::DAF‐16::6xHis::GFP, pRF4],* DH26 *fer‐15(b26ts) II*, MQD1319 *daf‐16(mu86) I; fer‐15(b26ts) II *and MQD1586 *hqEx476[hsp‐16.2p::nCherry; dod‐3p::gfp; mtl‐1p::bfp, unc‐119(+)]*; *unc‐119(ed3) III* EQ1137 *daf‐16(mu86) I; iqIs79[pAH123(daf‐16p::daf‐16::gfp^wt^, pRF4];* EQ1079 *daf‐16(mu86) I; iqIs83[pAH144(daf‐16p::daf‐16^4KR, K246+251+373+377R^)::gfp, pRF4]*; EQ1070 *daf‐16(mu86) I; iqIs83[pAH144(daf‐16p::daf‐16^4KR, K246+251+373+377R^)::gfp,pRF4]*

To generate transgenic animals, a plasmid DNA mix was microinjected into the gonad using standard method. The plasmid DNA mix consisted of 30 ng/µl of the indicated plasmid and 80 ng/µl of the pRF4 or 10 ng/µl of the pCFJ151. The UV irradiation was used to integrate the extra‐chromosomal DNA into the genome. The integrated strains were further backcrossed 6X to CF1037 *daf‐16(mu86) I*. MQD1319 was generated from CF1038 *daf‐16(mu86) I* and DH26 *fer‐15(b26ts) II *and backcrossed to DH26 for four times.

### 4.2 DAF‐16 nuclear localization

For the effect of the culture temperature on DAF‐16::GFP localization, worms were cultured on standard nematode growth medium (NGM) plates. For ThT treatment, L4 larvae grown at 20°C were transferred to NGM plates containing 50 μM ThT (SIGMA, St. Louis, MO, USA) dissolved in ethanol. NGM plates with equal amount of ethanol served as the control. As soon as worms were removed from incubation, they were mounted on slides and imaged immediately. The images were taken using a Zeiss Axio Imager M1 microscope at 400‐fold magnification. For the ThT experiments, DAF‐16::GFP fluorescent images were taken using the YFP channel to avoid interference of ThT fluorescence. All other fluorescent images of DAF‐16::GFP nuclear were taken using the GFP channel.

For the effect of the culture temperature and ThT on DAF‐16::GFP localization, each worm was given a score: cytoplasmic, 0; weakly nuclear, 1; strongly nuclear, 2.

For the analysis of nuclear localization in KR mutant worms, GFP localization was then analyzed using an Olympus BX61 fluorescent microscope on adult day 1 and day 5. An animal was scored as having nuclear GFP if more than one intestinal nucleus contained enriched GFP signal. The quantification of DAF‐16::GFP localization was described previously in (Chiang, Ching, Lee, Mousigian, & Hsu, 2012).

### 4.3 Quantitative real‐time PCR

Total RNAs were extracted from WT or *daf‐16(mu86)* worms of the indicated age using TRIzol (INVITROGEN, Grand Island, NY, USA), followed by the removal of contaminant DNA using DNase I. cDNAs were synthesized from total RNA templates using a reverse transcription kit (TAKARA, Kusatsu, Shiga, Japan). qPCR was carried out on an ABI 7500 Fast real‐time PCR system using a TAKARA real‐time PCR kit (SYBR Premix Ex TaqTM II). *pmp‐3 *was used as the internal control. The qPCR primers were as follows:

*mtl‐1 *[TGAGGAGGCCAGTGAGAAAAA]/[GCTCTGCACAATGACAGTTTGC];

*ftn‐1* [TGACGCGCACTTGACAAATTA]/[TGTAGCGAGCAAATTCATTGATC];

*dod‐6* [CTCAAGACCGTCGCCCTCTA]/[TCAGCATCAGCGCAAGCA];

*lys‐7* [CATTCGGCATCAGTCAAGGTT]/[GCAGGCTCCGCAATGACTT];

*hsp‐16.2* [TACGCTATCAATCCAAGGAGAAC]/[GAAGCAACTGCACCAACATC];

*pmp‐3* [GAATGGAATTGTTTCACGGAATGC]/[CTCTTCGTGAAGTTCCATAACACGATG].

### 4.4 RNA sequencing and data analysis

Transcriptome analyses of *fer‐15(b26ts)* and *daf‐16(mu86); fer‐15(b26ts*) worms were carried out in three biological replicates. Worms were grown on high‐growth (HG) plates supplemented with OP50 bacteria at 25°C and harvested on adult day 1 through day 7. RNA quality was evaluated on a Bioanalyzer 2100 instrument (Agilent, Santa Clara, CA). Sequencing libraries were prepared following the protocol of the NEBNext Ultra RNA library Prep Kit (NEB) and sequenced on an Illumina HiSeq X Ten platform in the paired‐end mode (2 × 150 bp) through the service provided by Bionova. An average of 25 M clean reads were generated for each replicate. RNA‐Seq reads were aligned to the *C. elegans* reference genome (ws235) using HISAT2 (v2.0.4; Kim, Langmead, & Salzberg, 2015) with default parameters. Gene‐level read counts were calculated using HTSeq (v0.6.1p1; Anders, Pyl, & Huber, 2015) based on the Ensembl gene annotation v85. DEseq2 (v1.18.1) was used for data normalization (Love, Huber, & Anders, 2014).

Hieratical clustering of samples by Spearman's correlation of gene expression was adapted to examine the quality of the data. Five outliers (rep 3 of *fer‐15* on Day 2/3/6, rep 3 of *daf‐16; fer‐15* on Day 2, and rep 2 of *daf‐16; fer‐15* on Day 5) were removed from further analysis.

Statistical analysis of differential expression was performed using the *nbinomWaldTest* in DESeq2 package. Genes with an adjusted *p*‐value <0.05 were defined as differentially expressed genes (DEGs).

In defining age‐dependent DAF‐16 targets, the day‐6 and day‐7 DEGs were filtered by requiring an adjusted *p*‐value <0.1. Class I and II DAF‐16 targets under reduced IIS are from Tepper et al. (2013).

### 4.5 Enrichment analysis

Gene Ontology (GO) and KEGG pathway databases were downloaded from the KOBAS website (Xie et al., 2011). The enrichment analysis was done using a hypergeometric test, and the *p‐*values were adjusted using the BH (Benjamini & Hochberg, 1995) method.

### 4.6 K‐means clustering and data normalization

The normalized read counts of each gene across all samples were first transformed to Z‐Score (mean centered and scaled to the variance). K‐means clustering was performed using the *kmeans* function in R with the settings of 6 clusters and a maximum of 1,000 iterations.

### 4.7 Fluorescence transcriptional reporter assay

All fluorescent images in Figure 5 were taken using a Zeiss Axio Imager M1 microscope at 100× magnification. Each image was taken as a whole object, and the fluorescence intensity was calculated with background subtraction using ImageJ.

### 4.8 RNAi

RNAi assays were performed at 20°C using the feeding method as previously described (Tao et al., 2013). 50 μM FudR was added into the RNAi plate to prevent the contamination of progeny. Worms were fed RNAi bacteria from adult day 1. The RNAi bacterial strain of *daf‐16* was from the Ahringer RNAi library, and other RNAi bacterial strains were from the Vidal RNAi library. The *Escherichia coli* strain HT115 transfected with L4440 (empty vector) was used as control.

## AUTHOR CONTRIBUTIONS

S.‐T.L. contributed to conception and design, acquisition of data, analysis and interpretation of data, and drafting and revising the article. H.‐Q.Z. involved in conception and design, analysis and interpretation of data, and drafting and revising the article. P.Z. contributed to conception and design, and acquisition of data. C.‐Y.L. involved in acquisition of data. Y.‐P.Z. contributed to acquisition of data. A.‐L.H. involved in interpretation of data. M.‐Q.D. contributed to conception and design, analysis and interpretation of data, and drafting and revising the article.

## DATA AVAILABILITY

All the FASTQ files for RNA‐Seq from this study will be submitted to the NCBI Gene Expression Omnibus (GEO) Database under accession number GSE122892.
