# Supplementary material for: DAF‐16 stabilizes the aging transcriptome and is activated in mid‐aged Caenorhabditis elegans to cope with internal stress
Source: Aging Cell. 2019 Feb 17;18(3):e12896. doi: 10.1111/acel.12896 (PMC6516157; doi:10.1111/acel.12896)

Figure S1

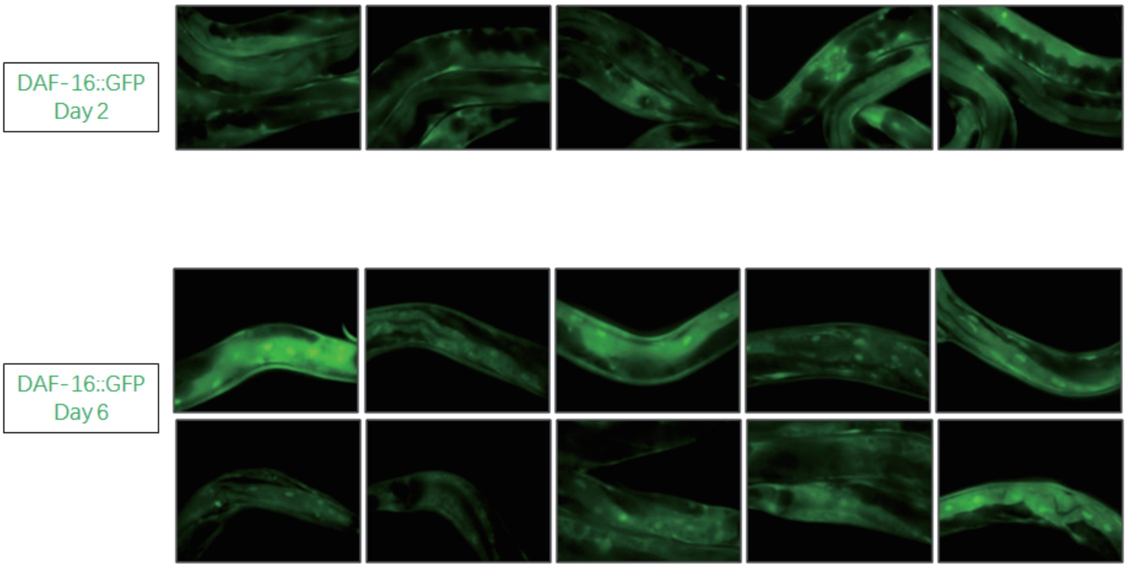

Figure S2

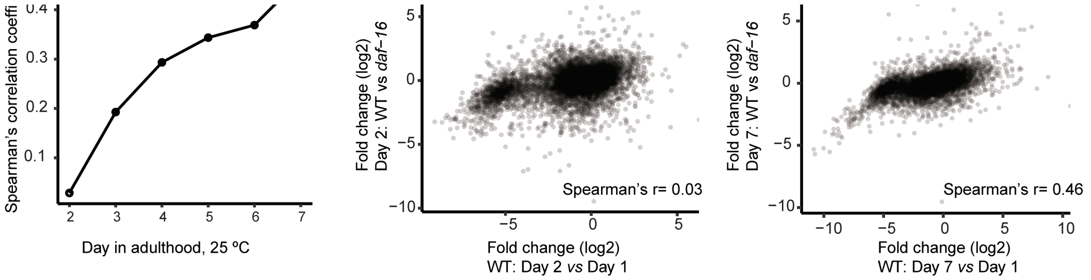

Figure S3

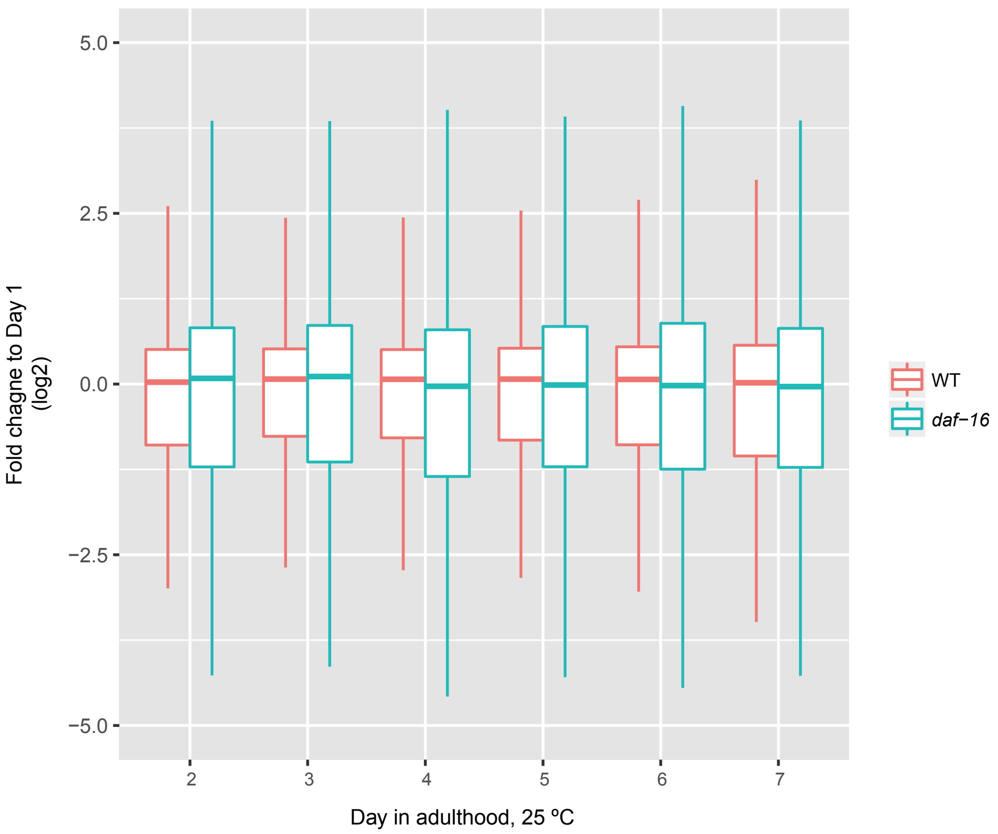

Figure S4

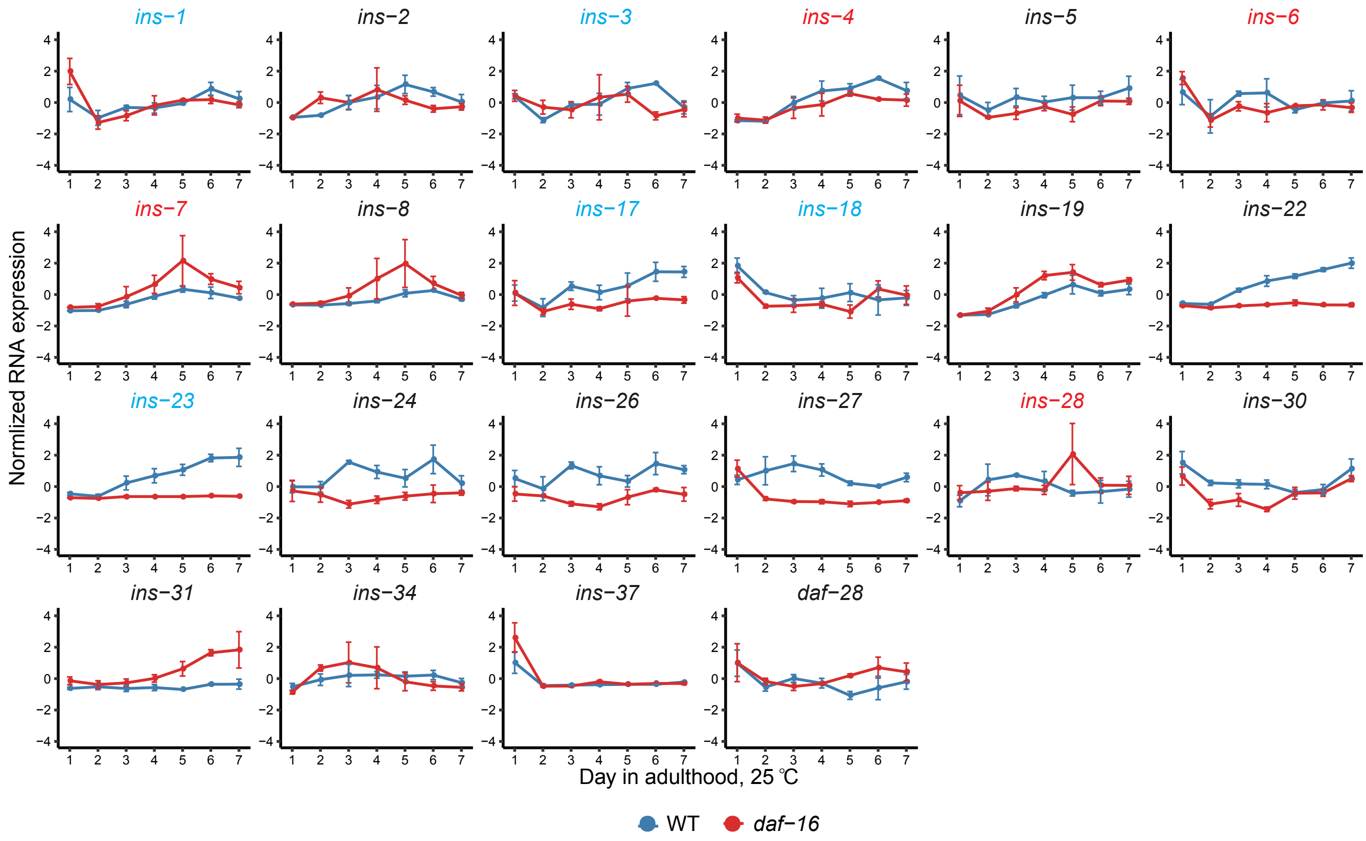

Figure S5

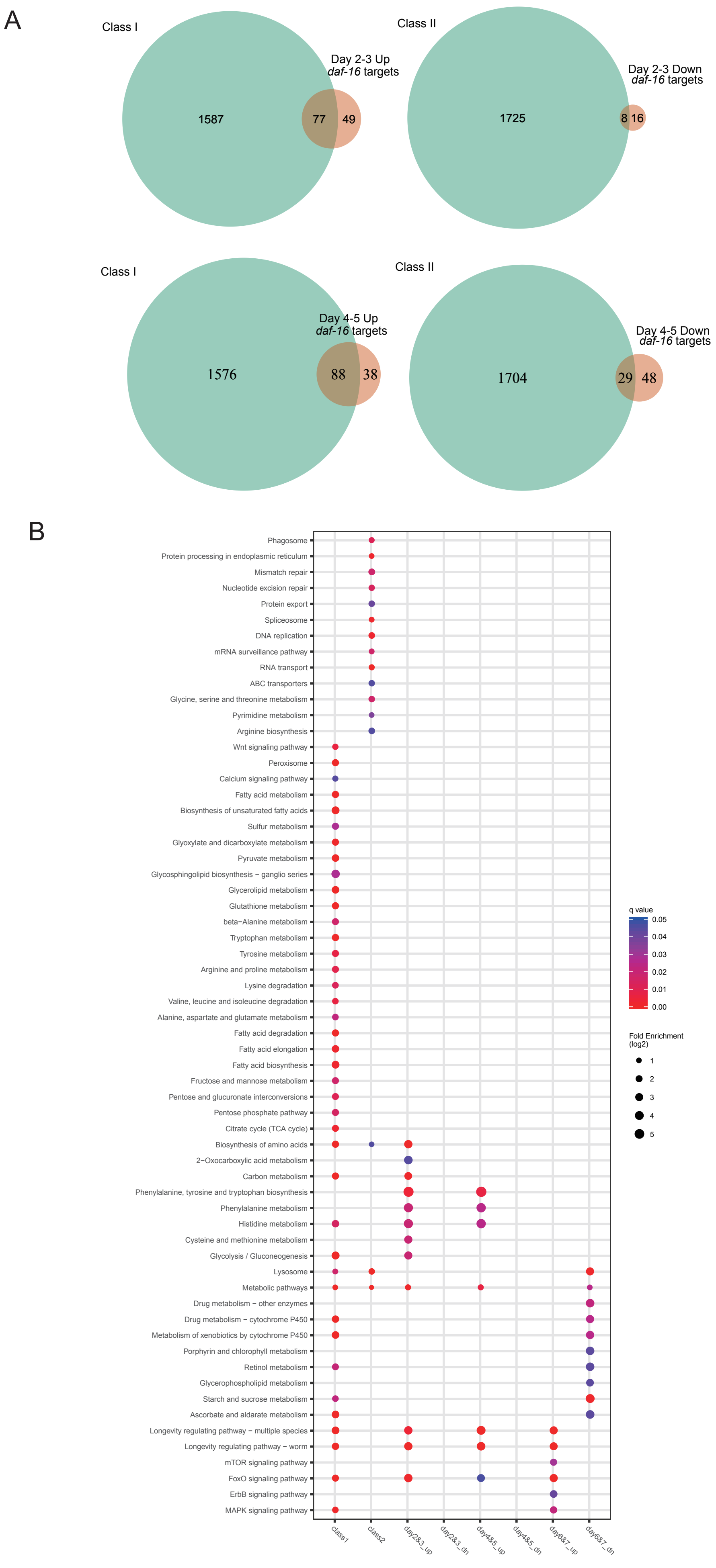
A
Class I
Day 2-3 Up
daf-16 targets
1587
77
49
Class II
Day 2-3 Down
daf-16 targets
1725
8
16
Class I
Day 4-5 Up
daf-16 targets
1576
88
38
Class II
Day 4-5 Down
daf-16 targets
1704
29
48
B
Phagosome
Protein processing in endoplasmic reticulum
Mismatch repair
Nucleotide excision repair
Protein export
Spliceosome
DNA replication
mRNA surveillance pathway
RNA transport
ABC transporters
Glycine, serine and threonine metabolism
Pyrimidine metabolism
Arginine biosynthesis
Wnt signaling pathway
Peroxisome
Calcium signaling pathway
Fatty acid metabolism
Biosynthesis of unsaturated fatty acids
Sulfur metabolism
Glyoxylate and dicarboxylate metabolism
Pyruvate metabolism
Glycosphingolipid biosynthesis − ganglio series
Glycerolipid metabolism
Glutathione metabolism
beta−Alanine metabolism
Tryptophan metabolism
Tyrosine metabolism
Arginine and proline metabolism
Lysine degradation
Valine, leucine and isoleucine degradation
Alanine, aspartate and glutamate metabolism
Fatty acid degradation
Fatty acid elongation
Fatty acid biosynthesis
Fructose and mannose metabolism
Pentose and glucuronate interconversions
Pentose phosphate pathway
Citrate cycle (TCA cycle)
Biosynthesis of amino acids
2−Oxocarboxylic acid metabolism
Carbon metabolism
Phenylalanine, tyrosine and tryptophan biosynthesis
Phenylalanine metabolism
Histidine metabolism
Cysteine and methionine metabolism
Glycolysis / Gluconeogenesis
Lysosome
Metabolic pathways
Drug metabolism − other enzymes
Drug metabolism − cytochrome P450
Metabolism of xenobiotics by cytochrome P450
Porphyrin and chlorophyll metabolism
Retinol metabolism
Glycerophospholipid metabolism
Starch and sucrose metabolism
Ascorbate and aldarate metabolism
Longevity regulating pathway − multiple species
Longevity regulating pathway − worm
mTOR signaling pathway
FoxO signaling pathway
ErbB signaling pathway
MAPK signaling pathway
class1
class2
day2&3_up
day2&3_dn
day4&5_up
day4&5_dn
day6&7_up
day6&7_dn
q value
0.05
0.04
0.03
0.02
0.01
0.00
Fold Enrichment
(log2)
1
2
3
4
5

Figure S6

A

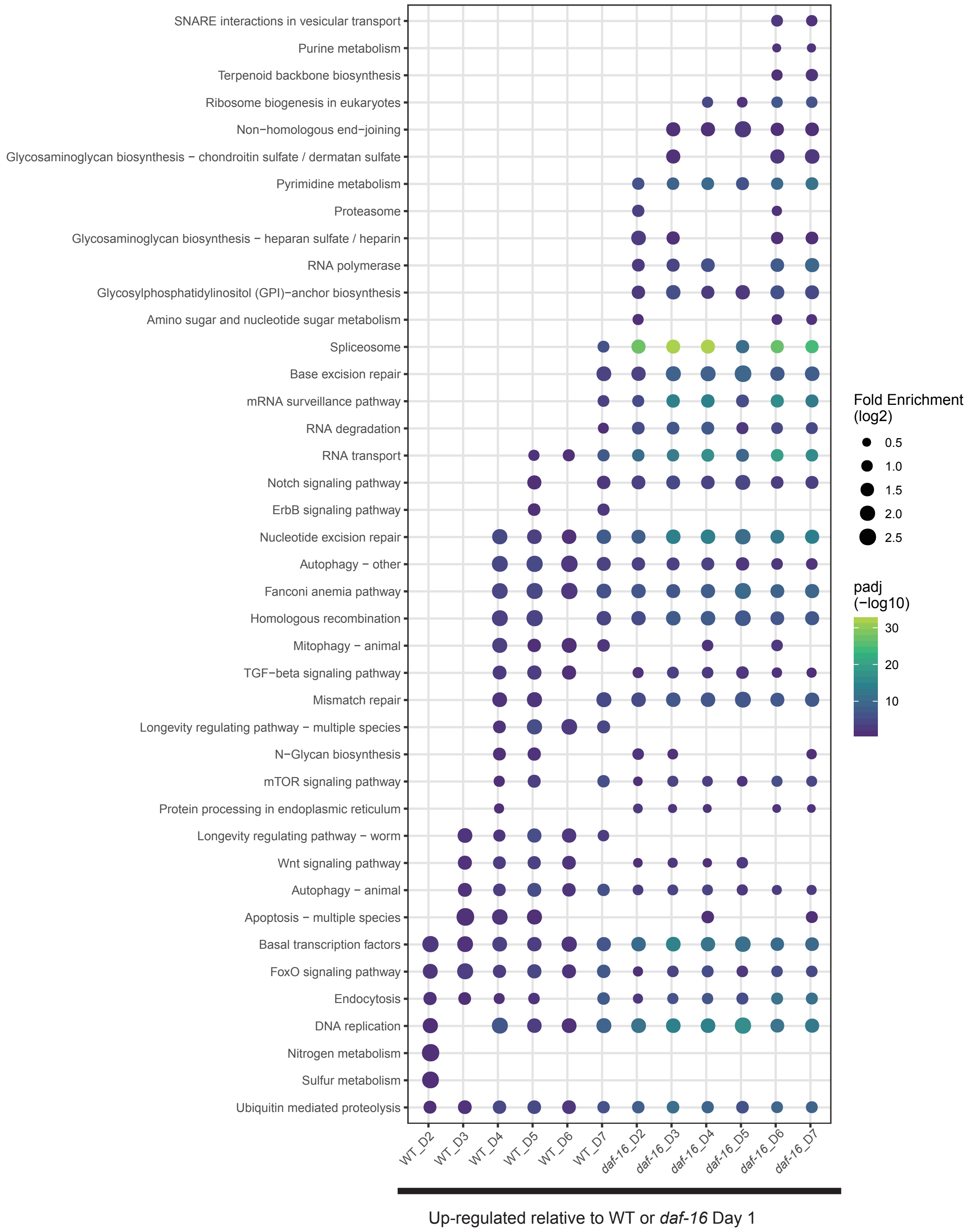

SNARE interactions in vesicular transport
Purine metabolism
Terpenoid backbone biosynthesis
Ribosome biogenesis in eukaryotes
Non−homologous end−joining
Glycosaminoglycan biosynthesis − chondroitin sulfate / dermatan sulfate
Pyrimidine metabolism
Proteasome
Glycosaminoglycan biosynthesis − heparan sulfate / heparin
RNA polymerase
Glycosylphosphatidylinositol (GPI)−anchor biosynthesis
Amino sugar and nucleotide sugar metabolism
Spliceosome
Base excision repair
mRNA surveillance pathway
RNA degradation
RNA transport
Notch signaling pathway
ErbB signaling pathway
Nucleotide excision repair
Autophagy − other
Fanconi anemia pathway
Homologous recombination
Mitophagy − animal
TGF−beta signaling pathway
Mismatch repair
Longevity regulating pathway − multiple species
N−Glycan biosynthesis
mTOR signaling pathway
Protein processing in endoplasmic reticulum
Longevity regulating pathway − worm
Wnt signaling pathway
Autophagy − animal
Apoptosis − multiple species
Basal transcription factors
FoxO signaling pathway
Endocytosis
DNA replication
Nitrogen metabolism
Sulfur metabolism
Ubiquitin mediated proteolysis
WT_D2
WT_D3
WT_D4
WT_D5
WT_D6
WT_D7
daf-16_D2
daf-16_D3
daf-16_D4
daf-16_D5
daf-16_D6
daf-16_D7
Fold Enrichment
(log2)
0.5
1.0
1.5
2.0
2.5
padj
(−log10)
30
20
10
Up-regulated relative to WT or daf-16 Day 1

B

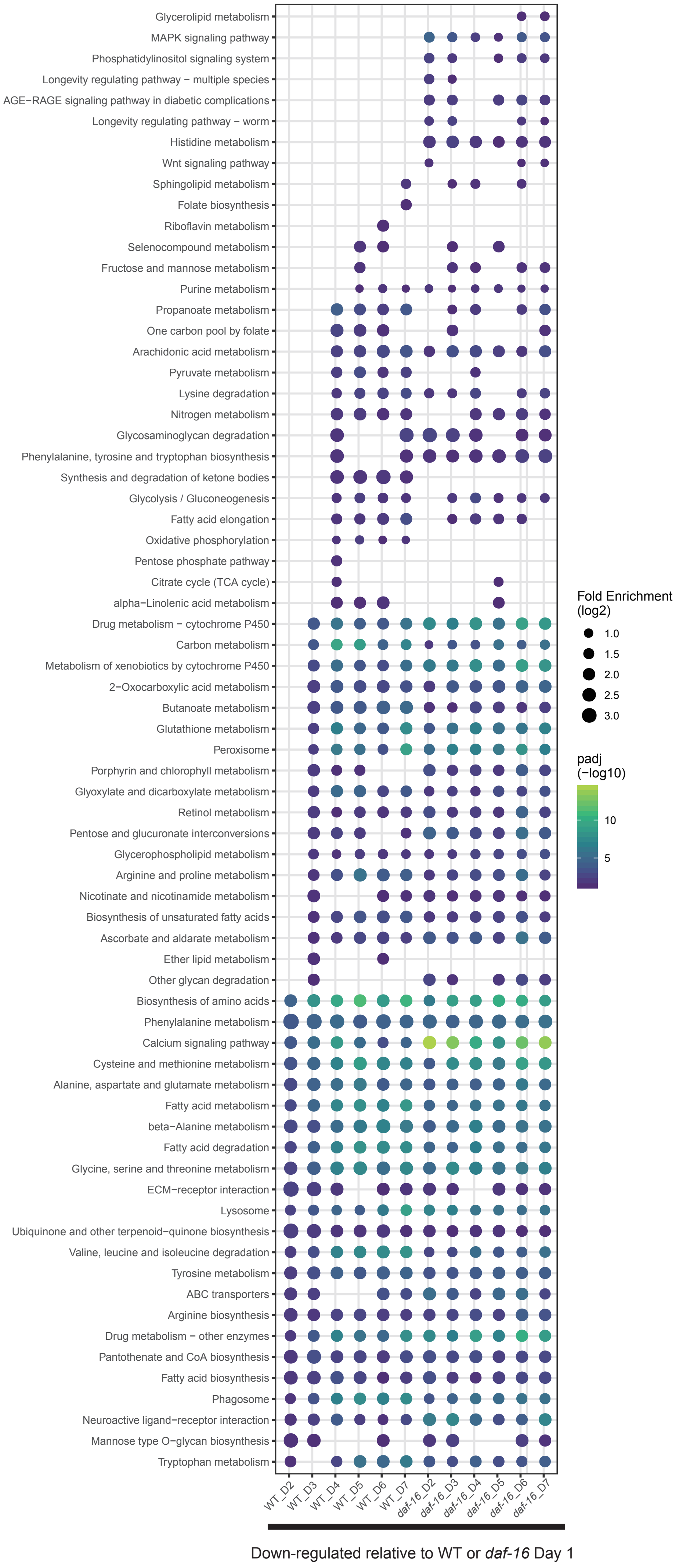
Glycerolipid metabolism
MAPK signaling pathway
Phosphatidylinositol signaling system
Longevity regulating pathway − multiple species
AGE−RAGE signaling pathway in diabetic complications
Longevity regulating pathway − worm
Histidine metabolism
Wnt signaling pathway
Sphingolipid metabolism
Folate biosynthesis
Riboflavin metabolism
Selenocompound metabolism
Fructose and mannose metabolism
Purine metabolism
Propanoate metabolism
One carbon pool by folate
Arachidonic acid metabolism
Pyruvate metabolism
Lysine degradation
Nitrogen metabolism
Glycosaminoglycan degradation
Phenylalanine, tyrosine and tryptophan biosynthesis
Synthesis and degradation of ketone bodies
Glycolysis / Gluconeogenesis
Fatty acid elongation
Oxidative phosphorylation
Pentose phosphate pathway
Citrate cycle (TCA cycle)
alpha−Linolenic acid metabolism
Drug metabolism − cytochrome P450
Carbon metabolism
Metabolism of xenobiotics by cytochrome P450
2−Oxocarboxylic acid metabolism
Butanoate metabolism
Glutathione metabolism
Peroxisome
Porphyrin and chlorophyll metabolism
Glyoxylate and dicarboxylate metabolism
Retinol metabolism
Pentose and glucuronate interconversions
Glycerophospholipid metabolism
Arginine and proline metabolism
Nicotinate and nicotinamide metabolism
Biosynthesis of unsaturated fatty acids
Ascorbate and aldarate metabolism
Ether lipid metabolism
Other glycan degradation
Biosynthesis of amino acids
Phenylalanine metabolism
Calcium signaling pathway
Cysteine and methionine metabolism
Alanine, aspartate and glutamate metabolism
Fatty acid metabolism
beta−Alanine metabolism
Fatty acid degradation
Glycine, serine and threonine metabolism
ECM−receptor interaction
Lysosome
Ubiquinone and other terpenoid−quinone biosynthesis
Valine, leucine and isoleucine degradation
Tyrosine metabolism
ABC transporters
Arginine biosynthesis
Drug metabolism − other enzymes
Pantothenate and CoA biosynthesis
Fatty acid biosynthesis
Phagosome
Neuroactive ligand−receptor interaction
Mannose type O−glycan biosynthesis
Tryptophan metabolism
WT_D2
WT_D3
WT_D4
WT_D5
WT_D6
WT_D7
daf-16_D2
daf-16_D3
daf-16_D4
daf-16_D5
daf-16_D6
daf-16_D7
Fold Enrichment
(log2)
1.0
1.5
2.0
2.5
3.0
padj
(−log10)
10
5
Down-regulated relative to WT or daf-16 Day 1

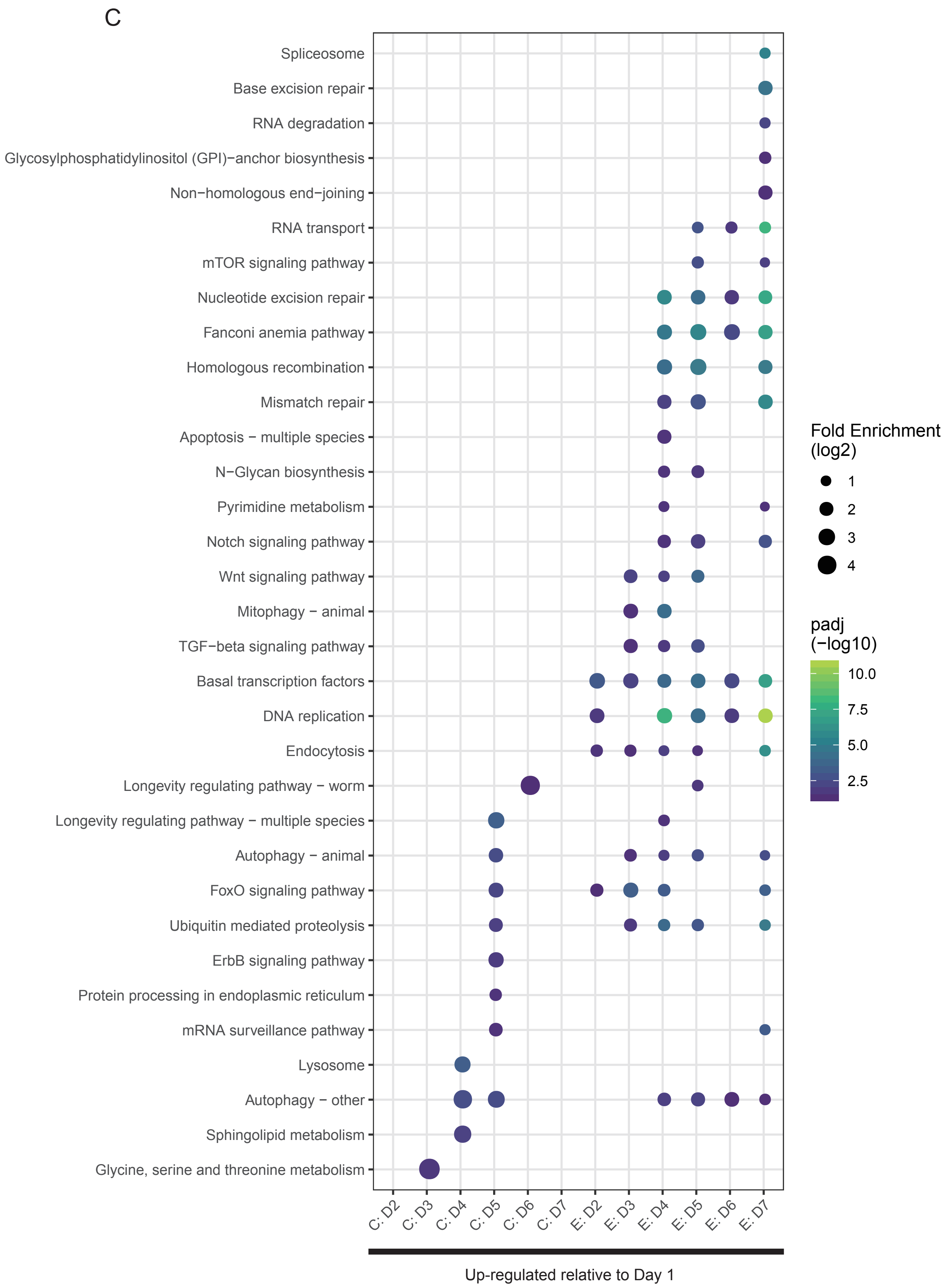
C
Spliceosome
Base excision repair
RNA degradation
Glycosylphosphatidylinositol (GPI)−anchor biosynthesis
Non−homologous end−joining
RNA transport
mTOR signaling pathway
Nucleotide excision repair
Fanconi anemia pathway
Homologous recombination
Mismatch repair
Apoptosis − multiple species
N−Glycan biosynthesis
Pyrimidine metabolism
Notch signaling pathway
Wnt signaling pathway
Mitophagy − animal
TGF−beta signaling pathway
Basal transcription factors
DNA replication
Endocytosis
Longevity regulating pathway − worm
Longevity regulating pathway − multiple species
Autophagy − animal
FoxO signaling pathway
Ubiquitin mediated proteolysis
ErbB signaling pathway
Protein processing in endoplasmic reticulum
mRNA surveillance pathway
Lysosome
Autophagy − other
Sphingolipid metabolism
Glycine, serine and threonine metabolism
C: D2
C: D3
C: D4
C: D5
C: D6
C: D7
E: D2
E: D3
E: D4
E: D5
E: D6
E: D7
Up-regulated relative to Day 1
Fold Enrichment (log2)
1
2
3
4
padj (−log10)
10.0
7.5
5.0
2.5

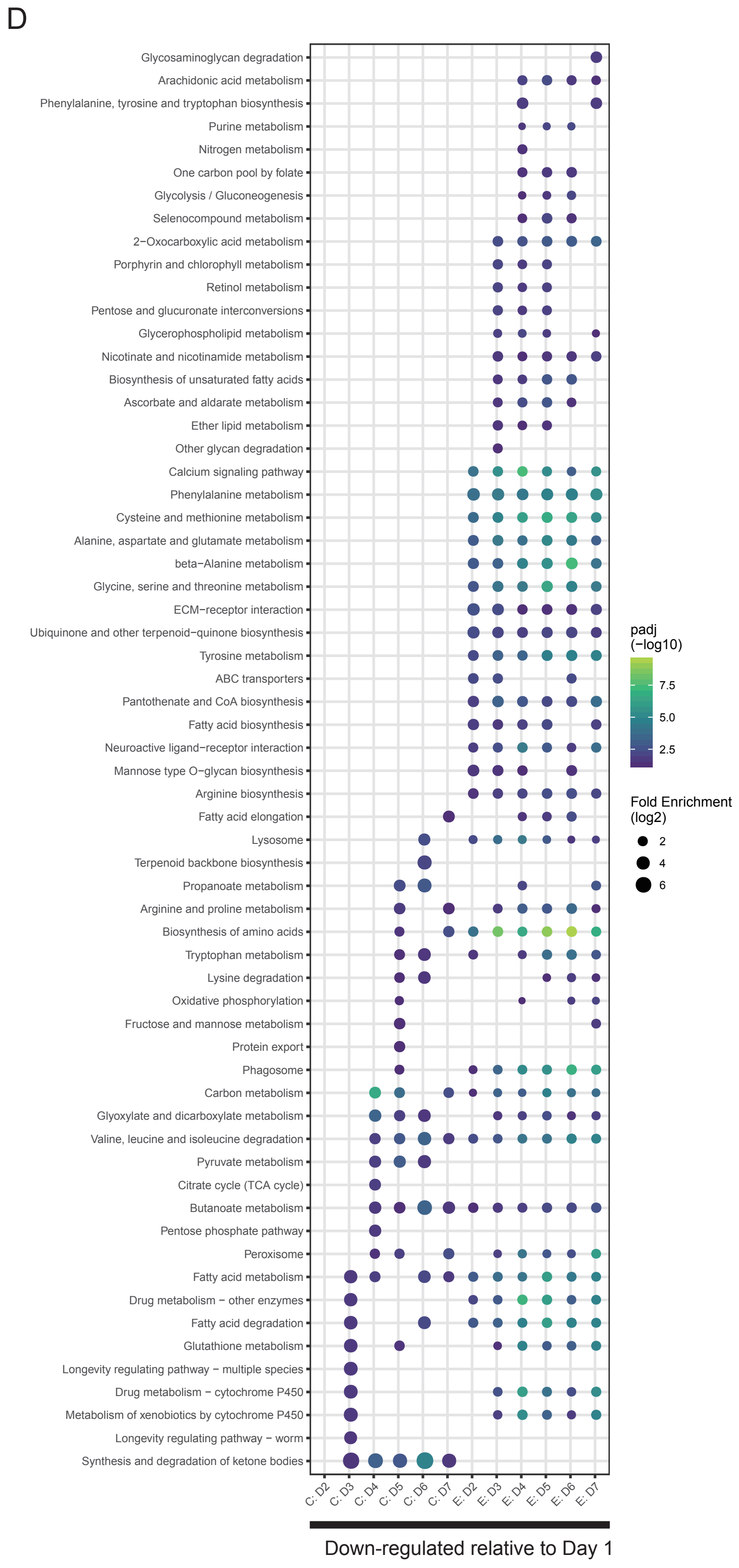
D
Glycosaminoglycan degradation
Arachidonic acid metabolism
Phenylalanine, tyrosine and tryptophan biosynthesis
Purine metabolism
Nitrogen metabolism
One carbon pool by folate
Glycolysis / Gluconeogenesis
Selenocompound metabolism
2−Oxocarboxylic acid metabolism
Porphyrin and chlorophyll metabolism
Retinol metabolism
Pentose and glucuronate interconversions
Glycerophospholipid metabolism
Nicotinate and nicotinamide metabolism
Biosynthesis of unsaturated fatty acids
Ascorbate and aldarate metabolism
Ether lipid metabolism
Other glycan degradation
Calcium signaling pathway
Phenylalanine metabolism
Cysteine and methionine metabolism
Alanine, aspartate and glutamate metabolism
beta−Alanine metabolism
Glycine, serine and threonine metabolism
ECM−receptor interaction
Ubiquinone and other terpenoid−quinone biosynthesis
Tyrosine metabolism
ABC transporters
Pantothenate and CoA biosynthesis
Fatty acid biosynthesis
Neuroactive ligand−receptor interaction
Mannose type O−glycan biosynthesis
Arginine biosynthesis
Fatty acid elongation
Lysosome
Terpenoid backbone biosynthesis
Propanoate metabolism
Arginine and proline metabolism
Biosynthesis of amino acids
Tryptophan metabolism
Lysine degradation
Oxidative phosphorylation
Fructose and mannose metabolism
Protein export
Phagosome
Carbon metabolism
Glyoxylate and dicarboxylate metabolism
Valine, leucine and isoleucine degradation
Pyruvate metabolism
Citrate cycle (TCA cycle)
Butanoate metabolism
Pentose phosphate pathway
Peroxisome
Fatty acid metabolism
Drug metabolism − other enzymes
Fatty acid degradation
Glutathione metabolism
Longevity regulating pathway − multiple species
Drug metabolism − cytochrome P450
Metabolism of xenobiotics by cytochrome P450
Longevity regulating pathway − worm
Synthesis and degradation of ketone bodies
C: D2
C: D3
C: D4
C: D5
C: D6
C: D7
E: D2
E: D3
E: D4
E: D5
E: D6
E: D7
Down-regulated relative to Day 1
padj
(−log10)
7.5
5.0
2.5
Fold Enrichment
(log2)
2
4
6

Figure S7

DVE-1 targets

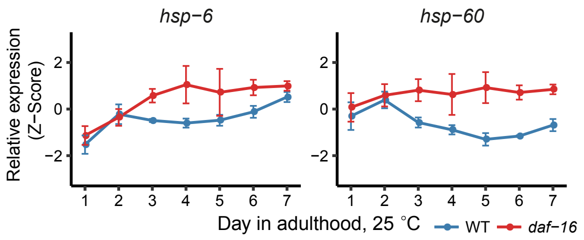

ELT-2 targets

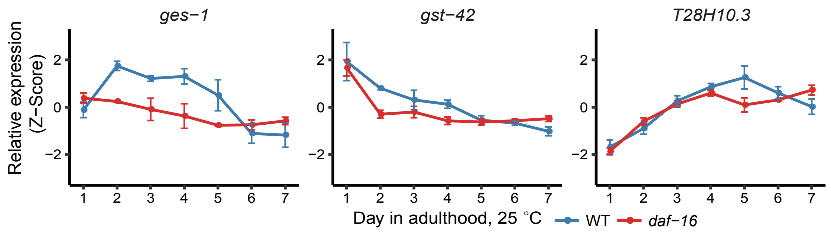

HLH-30 targets

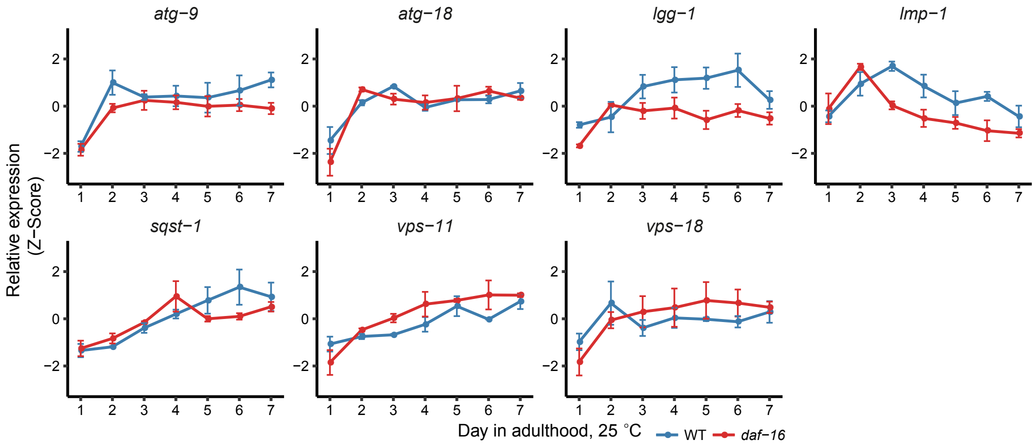

PHA-4 targets

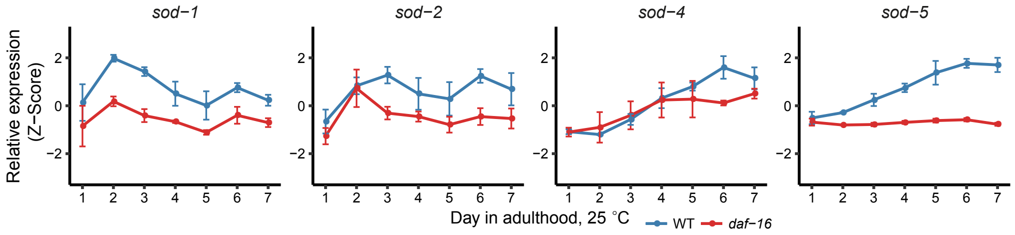

SKN-1 targets

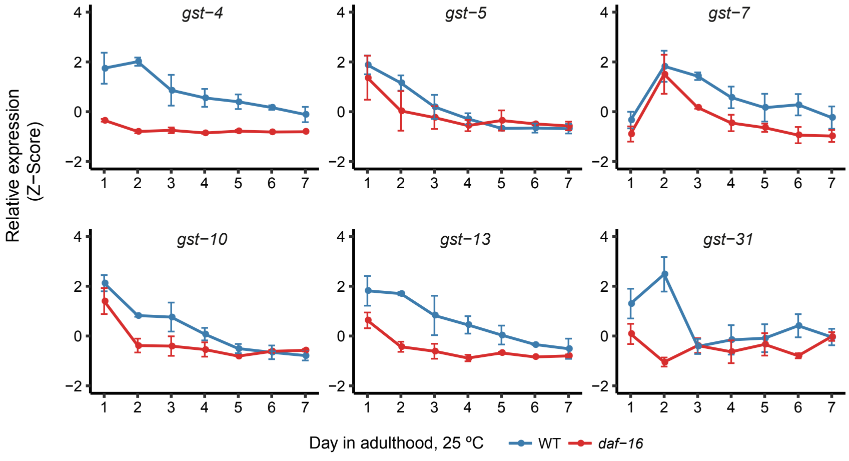

Supplement: Supplementary file 1 [file ACEL-18-e12896-s001.pdf]
